# Synovial fluid IL-16 and RANTES/CCL5 signals in early knee osteoarthritis: a pilot antibody-array study

**DOI:** 10.3389/fimmu.2026.1889340

**Published:** 2026-07-08

**Authors:** Bei Lin, Boyue Wu, Huihuang Chen, Wentao Lin, Dasheng Lin, Eryou Feng

**Affiliations:** Department of Orthopedic Surgery, Fujian Medical University Union Hospital, Fuzhou, China

**Keywords:** antibody array, CCL5/RANTES, cytokines, hypothesis-generating analysis, IL-16, osteoarthritis, pilot study, synovial fluid

## Abstract

**Background:**

Reliable molecular indicators for early osteoarthritis (OA) remain limited. Because synovial fluid (SF) reflects the intra-articular immune microenvironment, inflammatory signals may help describe preliminary features associated with early OA for future validation. This pilot, hypothesis-generating study used a high-throughput antibody array to profile SF cytokines across radiographic groups of knee OA.

**Methods:**

Sixteen participants were included: a K–L grade 0 non-radiographic comparator group (K0, n = 4), early OA (K1; Kellgren–Lawrence grade 1–2, n = 6), and late OA (K3; grade 3–4, n = 6). Forty inflammatory proteins were measured using the RayBiotech Human Inflammation Antibody Array.

**Results:**

In K1 versus K0, IL-16 [fold change (FC) = 15.79; logFC = 3.98; approximate *post hoc* uncertainty interval for logFC, 2.21 to 5.75; adj.P.Val = 0.027] and RANTES/CCL5 (FC = 11.83; logFC = 3.56; approximate *post hoc* uncertainty interval for logFC, −1.23 to 8.36; adj. P.Val = 0.027) were the only proteins that remained significant after Benjamini–Hochberg correction. PCA and heatmap analyses based on selected proteins were used only as visualization of selected signals and not as evidence of diagnostic discrimination. In K3 versus K1, seven proteins showed exploratory downregulation based on raw P values but did not survive multiple-testing correction. GO/KEGG analyses were therefore interpreted as supplementary hypothesis generation only.

**Conclusions:**

In this very small pilot cohort, IL-16 and RANTES/CCL5 showed array-based inflammatory signals associated with early OA after multiple-testing correction. The findings do not constitute validation of a clinical marker, and no diagnostic claim is made because ELISA confirmation, ROC analysis, and an independent validation cohort were not available. These data should therefore be regarded as preliminary discovery results, and larger, adequately powered studies with orthogonal quantitative validation are required before clinical translation can be considered.

## Background

Osteoarthritis (OA) is the most common degenerative joint disease worldwide, affecting more than 300 million people and representing a major cause of disability and reduced quality of life among middle-aged and older adults (1). Despite progress in clinical management, early identification and characterization remain challenging. Reliable molecular indicators of early disease and disease-modifying therapies are still lacking, and many patients are diagnosed only after substantial structural damage has occurred (2). The concept and classification of early knee OA continue to evolve and remain heterogeneous (3, 4).

OA was long considered primarily a mechanical wear-and-tear disorder. Increasing evidence now indicates that low-grade chronic inflammation and synovitis contribute to OA pathogenesis (5). Synovial fluid (SF) directly reflects the intra-articular microenvironment and contains a broad range of cytokines, chemokines, and growth factors. SF may therefore provide an important source of samples for discovering inflammatory signals associated with OA radiographic groups (6, 7). Several studies have reported that pro-inflammatory mediators, including IL-1β, TNF-α and IL-6, are elevated in OA synovial fluid and are associated with disease severity (8, 9).

OA is not a homogeneous or static process. Its pathological features may differ across radiographic groups. Early OA is often characterized by active synovitis, whereas late OA is frequently dominated by cartilage and subchondral bone destruction (10). This radiographic-group heterogeneity suggests that the intra-articular immune microenvironment may also differ across groups. However, systematic cross-sectional comparisons of SF cytokine profiles across OA radiographic groups remain limited, although several SF cytokine-profiling studies have linked local inflammatory mediators to radiographic severity, pain, disability, or early-versus-late OA differences (11–15).

Among selected group-associated mediators, interleukin-16 (IL-16) is a multifunctional cytokine with chemotactic and immunomodulatory properties. It recruits CD4+ T cells, monocytes, and eosinophils to sites of inflammation via CD4 (16). IL-16 has been implicated in rheumatoid arthritis (RA) and other autoimmune diseases (17), but its expression across OA radiographic groups in synovial fluid has not been fully characterized. RANTES, also known as CCL5, is a key CC chemokine with chemotactic activity for multiple immune cell types (18). Synovitis has been linked to chemokine expression in OA (19), but whether CCL5 differs across OA radiographic groups requires further evaluation.

Therefore, we used a high-throughput antibody array (RayBiotech Human Inflammation Antibody Array, QAH-INF-3) to quantify 40 inflammation-related proteins in synovial fluid from a K–L grade 0 non-radiographic comparator group, early OA, and late OA. The aim was to describe preliminary, pilot array-based inflammatory signals for future validation, not to establish clinically deployable markers.

## Materials and methods

### Study design and participants

This exploratory cross-sectional study was approved by the Ethics Committee of Fujian Medical University Union Hospital (approval number: 2025KY794) and was conducted in accordance with the Declaration of Helsinki. Participants were enrolled according to the American College of Rheumatology (ACR) criteria for knee OA and the Kellgren–Lawrence grading system (20, 21). Three groups were included: K–L grade 0 non-radiographic comparator group (K0, n = 4), early OA (K1, K–L grade 1–2, n = 6), and late OA (K3, K–L grade 3–4, n = 6). The available sample-registration file recorded early OA as K1–2 but did not preserve the exact split between K–L grade 1 and grade 2 participants; these grades were therefore pooled, because this was a pilot study with a very small sample size. The sample registration record identified the K0 samples as synovial fluid assigned to the K0 group. The available source records did not specify the clinical indication for K0 aspiration, whether the samples were obtained during arthroscopy, trauma evaluation, meniscal injury assessment, or another clinical setting, or whether MRI was used to exclude synovitis or meniscal pathology. Therefore, K0 should be interpreted as a small K–L grade 0 non-radiographic comparator group rather than healthy synovial fluid from population-based volunteers. Exclusion criteria were other arthritides, including RA, gout, or septic arthritis; intra-articular injections within the previous 3 months; systemic infection; and systemic autoimmune disease.

### Synovial fluid collection and processing

Synovial fluid was aspirated aseptically (2 mL–5 mL) from the suprapatellar pouch using an 18G needle. Samples were transported on ice and centrifuged at 2,500 rpm for 10 min at 4°C to remove cells and debris. Supernatants were aliquoted and stored at −80°C until analysis. The exact time from aspiration to freezing and the complete pre-assay freeze–thaw history were not documented in the available source records; this is acknowledged as a limitation.

### High-throughput antibody array

Inflammation-related proteins in synovial fluid were measured using the RayBiotech Human Inflammation Antibody Array (QAH-INF-3; RayBiotech Inc., Peachtree Corners, GA, USA), a sandwich-based array targeting 40 human inflammatory proteins. The lot number was not available in the service files provided to the authors. The full 40-protein panel is listed in **Supplementary Table 1**. Arrays were blocked for 1 h and incubated with synovial fluid diluted 1:2 at 4°C overnight. They were then incubated with biotin-labeled detection antibodies for 2 h at room temperature and Cy3-conjugated streptavidin for 1 h at room temperature, protected from light. Fluorescence was scanned at 532 nm using an InnoScan 300 scanner (Innopsys, Carbonne, France), and median fluorescence intensity (MFI) was extracted.

### Bioinformatics and statistical analysis

Signal intensities were extracted using ImageJ (NIH, USA). Background correction used blank spots, array-to-array normalization used internal positive controls, and technical replicate spots were averaged. The available service report indicates that samples were analyzed within the same antibody-array service run. Sample randomization, blinded image analysis, and formal batch-effect testing were not documented in the available source records. No additional spot-exclusion rule beyond the service-report output was applied by the authors, and below-detection values were not imputed for the reported proteins. Differential expression was analyzed in R using the limma package (22) with moderated t-statistics and empirical Bayes shrinkage. Analyses reported log2 fold change (logFC), raw P values, and Benjamini–Hochberg adjusted P values (23) (adj. P.Val). Differentially expressed proteins (DEPs) were defined as adj. P.Val <0.05 and fold change >1.2 or <0.83 (|logFC| >0.263). This fold-change threshold was prespecified before differential-expression filtering and followed the RayBiotech technical-report convention for exploratory antibody-array analyses; in this small cohort, it was used as a descriptive effect-size filter rather than as evidence of clinical relevance. Proteins meeting fold-change and raw P <0.05 thresholds but not adj. P.Val <0.05 were reported explicitly as exploratory findings.

Principal component analysis (PCA) and hierarchical clustering were conducted only as exploratory visualization of selected protein signals. PCA was not used to infer robust group discrimination or diagnostic performance because the cohort was very small, the number of input variables was limited, and the proteins were selected from the same comparison being visualized. Heatmaps used log2-normalized expression values that were row-scaled to Z-scores; for each protein, Z = (sample value − row mean)/row standard deviation. Heatmaps used Euclidean distance and complete linkage, consistent with RayBiotech reports. Exploratory GO and KEGG enrichment based on raw P proteins was treated as supplementary hypothesis generation and was not used to support mechanistic conclusions. ROC analysis was not performed because the study lacked an orthogonal validation assay, an independent validation cohort, and sufficient sample size for reliable diagnostic modeling. For baseline variables, age, BMI, VAS, and WOMAC were assessed using Kruskal–Wallis tests; sex was assessed using Fisher’s exact test because of small cell counts; and disease duration was summarized descriptively between K1 and K3. Pairwise comparisons were considered exploratory.

## Results

### Baseline characteristics

Baseline characteristics are shown in **Table 1**. Age (P = 0.127) and sex distribution (P = 0.684) did not differ significantly among groups, but these P values were treated descriptively because of the extremely small group sizes, and should not be interpreted as evidence of complete covariate balance. BMI tended to be higher in K3 than K0 (P = 0.087), and disease duration, VAS pain scores, and WOMAC total scores increased with disease severity. WOMAC total scores were 3.2 ± 1.8 in K0, 28.5 ± 6.3 in K1, and 62.4 ± 8.9 in K3 (P = 0.003). Data on smoking, metabolic syndrome, effusion grade, prior knee injury or surgery, detailed medication use, and NSAID exposure were not available for adjustment.

**Table 1:** Baseline characteristics of participants.

Variable	K–L 0 comparator (n = 4)	K1 (n = 6)	K3 (n = 6)	P value
Age (years)	52.3 ± 8.5	58.2 ± 7.4	63.8 ± 6.9	0.127
Sex (male/female)	2/2	2/4	2/4	0.684
BMI (kg/m²)	23.1 ± 2.4	25.2 ± 2.9	26.8 ± 3.1	0.087
Disease duration (months)	—	18.5 ± 12.3	72.4 ± 36.8	<0.001***
K–L grade	0	1–2	3–4	–
VAS pain score	0.5 ± 0.6	3.9 ± 1.6	6.8 ± 1.4	<0.001***
WOMAC total score	3.2 ± 1.8	28.5 ± 6.3	62.4 ± 8.9	0.003**

Data are presented as mean ± SD unless otherwise stated. Baseline P values are descriptive because the study was not powered to test baseline balance. Age, BMI, VAS, and WOMAC were assessed using Kruskal–Wallis tests; sex was assessed using Fisher’s exact test because of small cell counts. Disease duration was summarized descriptively between K1 and K3 because it was not applicable to K0. BMI, body mass index; K–L, Kellgren–Lawrence; VAS, visual analogue scale; WOMAC, Western Ontario and McMaster Universities Osteoarthritis Index. **P <0.01; ***P <0.001.

### Early OA vs K–L grade 0 non-radiographic comparator group (K1 vs K0)

**Figure 1:**
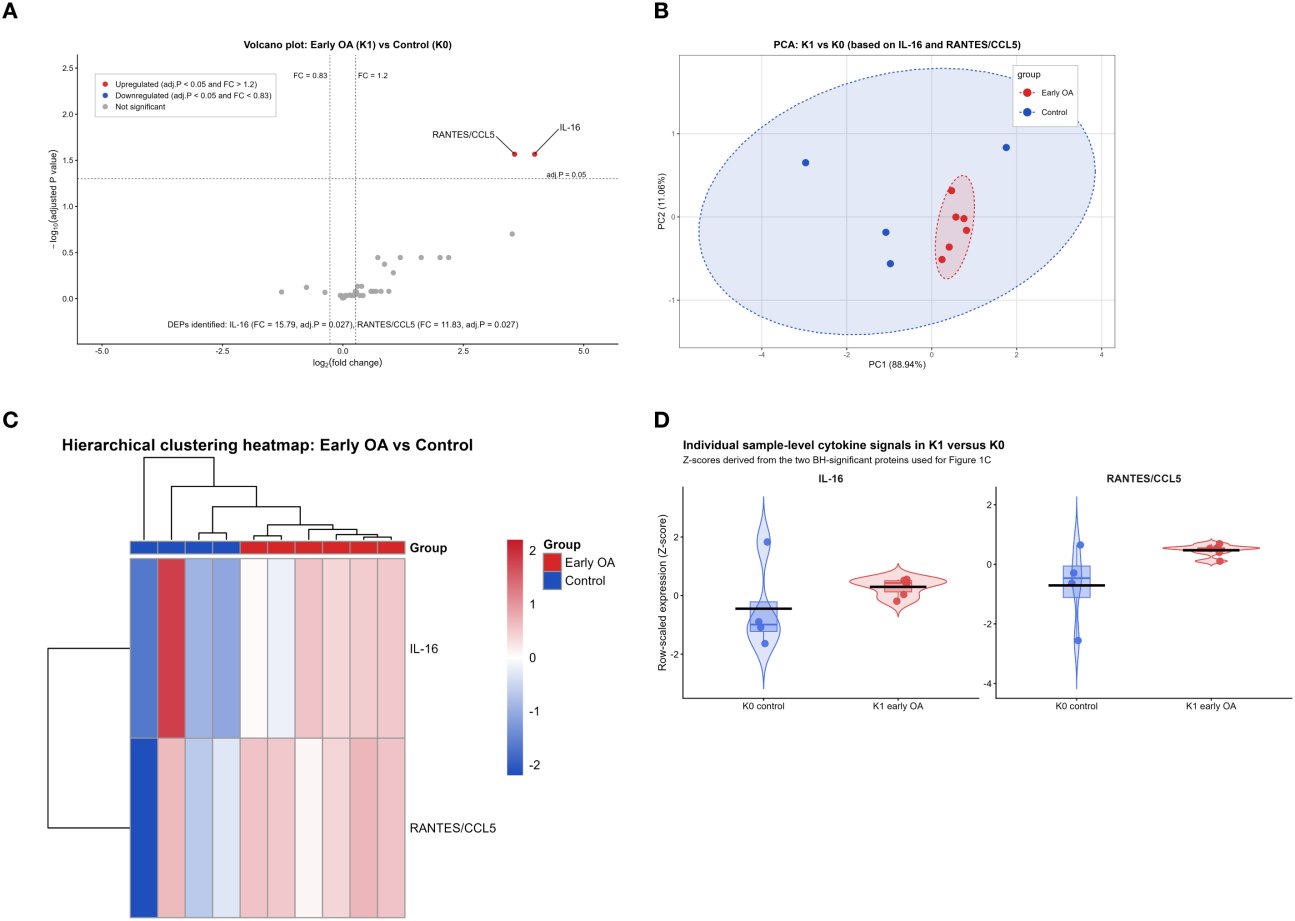
**(A)** Differential cytokine expression in early OA. Volcano plot of all 40 proteins comparing K1 with the K–L grade 0 non-radiographic comparator group (K0). Red dots indicate significantly upregulated differentially expressed proteins under the prespecified exploratory array threshold (adj. P.Val <0.05 and FC >1.2); gray dots indicate non-significant proteins. IL-16 and RANTES/CCL5 are labeled. **(B)** Exploratory PCA of K1 and K0 samples. Principal component analysis based on IL-16 and RANTES/CCL5 showed the visual distribution of selected signals between K1 (n = 6) and the K-L grade 0 comparator group (K0, n = 4) (PC1 = 88.94%; PC2 = 11.06%). This plot is descriptive and should not be interpreted as evidence of validated diagnostic discrimination. Because only two proteins selected from the same comparison entered the PCA model, the PCA output should be interpreted only as dimensionality-reduction visualization rather than a robust unsupervised classification analysis. **(C)** Hierarchical clustering of early OA-associated signals. Heatmap analysis using Euclidean distance and complete linkage visualized row-scaled Z-scores calculated from log2-normalized IL-16 and RANTES/CCL5 expression values between early OA and the K–L grade 0 comparator group. The clustering is exploratory. **(D)** Individual sample-level visualization of the two K1-versus-K0 signals. Violin and box plots with individual data points show row-scaled expression Z-scores for IL-16 and RANTES/CCL5 in the K–L grade 0 comparator group (K0, n = 4) and K1 early OA samples (n = 6). The plot is descriptive and is provided to show sample-level dispersion in this small pilot cohort.

Using the prespecified exploratory array threshold (adj. P.Val <0.05 and |logFC| > 0.263), two differentially expressed proteins were identified in K1 versus K0. Both were upregulated in early OA (**Table 2**; **Figure 1A**).

**Table 2:** Differentially expressed proteins in K1 vs K0 (adj. P.Val <0.05).

Protein	AveExpr K1	AveExpr K0	logFC	P value	adj. P	Fold change	Direction
IL-16	15.12	11.14	3.98	0.00077	0.027	15.79	Up
RANTES/CCL5	16.59	13.02	3.56	0.00136	0.027	11.83	Up

AveExp, average expression value after log2 transformation; logFC, log2 fold change; adj. P.Val, Benjamini–Hochberg-corrected P value; fold change = 2^logFC^. Approximate *post hoc* uncertainty intervals for logFC and fold change are provided in **Supplementary Table 2** and should be interpreted cautiously because of the very small sample size; they should not be regarded as formal inferential confidence intervals.

IL-16 showed the largest array-based change, increasing from AveExpr 11.14 in K0 to 15.12 in K1 (FC = 15.79; logFC = 3.98; approximate *post hoc* uncertainty interval for logFC, 2.21 to 5.75; approximate *post hoc* uncertainty interval for FC, 4.64 to 53.74; adj. P.Val = 0.027). RANTES/CCL5 increased from AveExpr 13.02 to 16.59 (FC = 11.83; logFC = 3.56; approximate *post hoc* uncertainty interval for logFC, −1.23 to 8.36; approximate *post hoc* uncertainty interval for FC, 0.42 to 329.60; adj. P.Val = 0.027). These were the only proteins that survived Benjamini–Hochberg correction, but the wide intervals, particularly for RANTES/CCL5, underscore the uncertainty caused by the small sample size. PCA based on these two proteins showed visual distribution of selected signals rather than independent evidence of discrimination (PC1 = 88.94%; PC2 = 11.06%; **Figure 1B**), and hierarchical clustering visualized group-associated expression patterns (**Figure 1C**). Individual sample-level distributions are shown in **Figure 1D** to make the small-sample dispersion transparent. These visual analyses should be interpreted cautiously because of the small sample size, selected input proteins, and low-dimensional input.

### Late OA vs K–L grade 0 non-radiographic comparator group (K3 vs K0)

IL-16 (FC = 6.50; P = 0.014) and RANTES/CCL5 (FC = 5.46; P = 0.019) remained upregulated in K3 versus K0, but neither protein remained significant after Benjamini–Hochberg correction (adj. P.Val = 0.380; **Table 3**). These results were therefore considered exploratory.

**Table 3:** Exploratory differentially expressed proteins in K3 vs K0 (raw P <0.05; not significant after correction).

Protein	AveExpr K3	AveExpr K0	logFC	P value	adj. P	Fold change	Direction
IL-16	13.84	11.14	2.70	0.014	0.380	6.50	Up
RANTES/CCL5	15.47	13.02	2.45	0.019	0.380	5.46	Up

Although raw P values were <0.05, neither protein achieved significance after Benjamini–Hochberg correction (adj. P.Val = 0.380). These results should be interpreted as exploratory.

The fold changes for both proteins were lower in K3 versus K0 than in K1 versus K0 (IL-16: FC 6.50 vs 15.79; CCL5: FC 5.46 vs 11.83). This cross-sectional observation is descriptive only and cannot establish longitudinal change, because K3-related comparisons did not survive multiple-testing correction.

### Late OA vs early OA (K3 vs K1)

**Figure 2:**
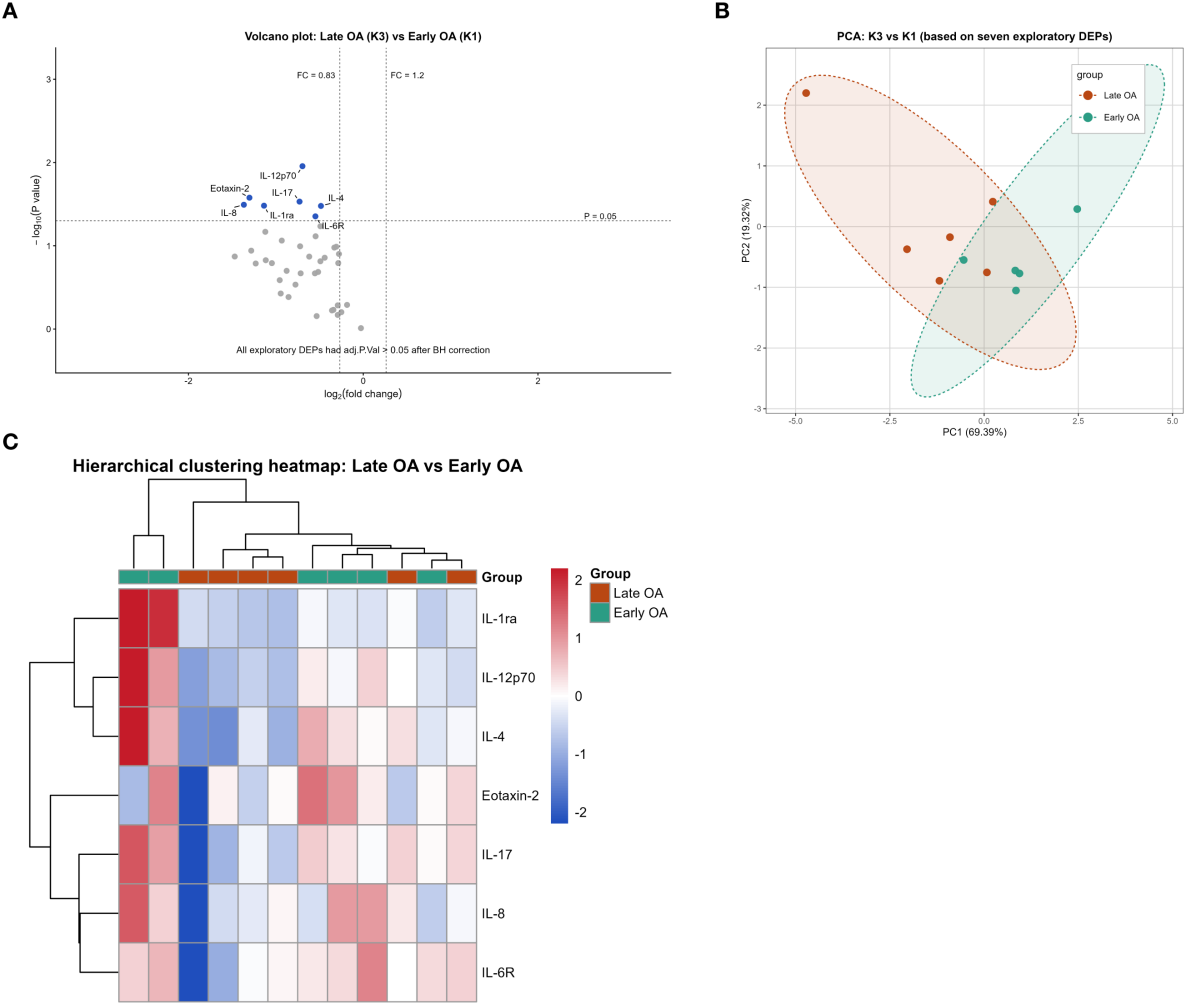
**(A)**. Exploratory cytokine changes in late OA. Volcano plot comparing K3 with K1. Seven proteins showed exploratory downregulation (raw P <0.05 and FC <0.83), but none passed Benjamini–Hochberg correction. **(B)**. Exploratory PCA of K3 and K1 samples. Principal component analysis based on the seven raw P exploratory proteins showed visual distribution of selected signals between K3 (n = 6) and K1 (n = 6) samples (PC1 = 69.39%; PC2 = 19.32%). This analysis is descriptive. Given the small sample size and the exploratory protein-selection strategy, this PCA should not be interpreted as evidence of robust subgroup discrimination. **(C)**. Hierarchical clustering of late versus early OA. Heatmap analysis using Euclidean distance and complete linkage visualized row-scaled Z-scores calculated from log2-normalized expression values for the seven raw P exploratory proteins in K3 versus K1.

Seven proteins were downregulated in K3 versus K1 under the exploratory threshold (raw P <0.05 and |logFC| >0.263): IL-8/CXCL8 (FC = 0.388), Eotaxin-2/CCL24 (FC = 0.406), IL-1ra (FC = 0.455), IL-17 (FC = 0.603), IL-12p70 (FC = 0.618), IL-4 (FC = 0.715), and IL-6R (FC = 0.684). None passed Benjamini–Hochberg correction (adj. P.Val = 0.222–0.253; **Table 4**; **Figure 2A**), so these findings should be interpreted as exploratory.

**Table 4:** Exploratory differentially expressed proteins in K3 vs K1 (raw P <0.05; not significant after correction).

Protein	AveExpr K3	AveExpr K1	logFC	P value	adj. P	Fold change	Direction
IL-8/CXCL8	12.13	13.50	−1.37	0.032	0.222	0.388	Down
Eotaxin-2/CCL24	13.26	14.57	−1.30	0.026	0.222	0.406	Down
IL-1ra	8.50	9.63	−1.14	0.033	0.222	0.455	Down
IL-17	7.98	8.70	−0.73	0.029	0.222	0.603	Down
IL-12p70	8.95	9.64	−0.70	0.011	0.222	0.618	Down
IL-4	9.92	10.40	−0.48	0.033	0.222	0.715	Down
IL-6R	15.39	15.94	−0.55	0.044	0.253	0.684	Down

All adj. P.Val values were ≥0.05; these results are exploratory and require validation. AveExpr, log2-transformed average expression; logFC, log2 fold change.

The seven raw P signals in K3 versus K1 were directionally consistent, but none remained significant after multiple-testing correction. IL-8, eotaxin-2/CCL24, IL-1ra, IL-17, IL-12p70, IL-6R, and IL-4, therefore represent raw P exploratory signals only. PCA based on these seven proteins showed visual distribution of selected signals (PC1 = 69.39%; PC2 = 19.32%; **Figure 2B**), and hierarchical clustering visualized a lower-expression pattern in late versus early OA (**Figure 2C**). These plots should not be used to infer robust classification.

### Cross-sectional expression patterns across OA radiographic groups

**Figure 3:**
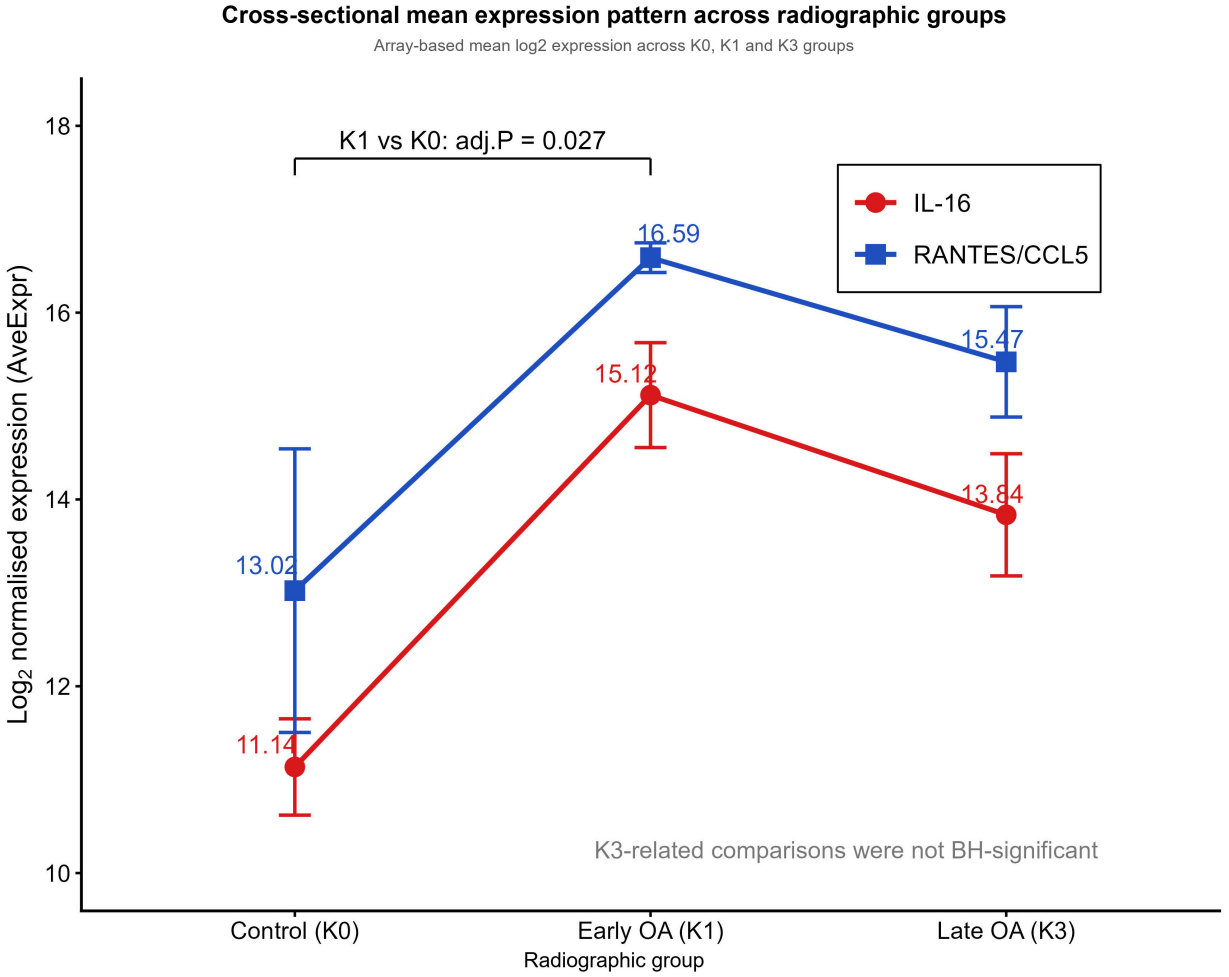
Cross-sectional mean expression pattern, not longitudinal trajectory. Line graph showing log2-normalized average expression (AveExpr) across the K–L grade 0 comparator group (K0), early OA (K1), and late OA (K3). Both cytokines showed higher mean values in K1 than in K0, whereas K3-related comparisons were not significant after Benjamini–Hochberg correction. This cross-sectional pattern should not be interpreted as longitudinal disease progression or temporal cytokine dynamics.

Across the three pairwise comparisons, IL-16 and RANTES/CCL5 showed a cross-sectional pattern with the highest mean expression in K1. Because the study was cross-sectional and because K3-related comparisons were not significant after multiple-testing correction, this observation should be considered hypothesis-generating and should not be interpreted as temporal change or longitudinal progression (**Figure 3**; **Table 5**).

**Table 5:** Summary of cross-sectional cytokine patterns across OA radiographic groups.

Cytokine	K0→K1 change	K1→K3 change	Overall pattern	Potential biological significance
IL-16	Significant upregulation (FC = 15.79, adj. P = 0.027)	Trending decrease (K3 vs. K1: logFC = −1.282, P = 0.114)	K1-high cross-sectional pattern	Hypothesis-generating early immune signal
RANTES/CCL5	Significant upregulation (FC = 11.83, adj. P = 0.027)	Trending decrease (K3 vs. K0: FC = 5.46, attenuated vs. K1)	K1-high cross-sectional pattern	Hypothesis-generating early chemokine signal
IL-8/CXCL8	Not significant	Raw-P exploratory decrease (FC = 0.388, P = 0.032)	Decreasing	Exploratory lower neutrophil-chemotaxis signal
IL-17	Not significant	Raw-P exploratory decrease (FC = 0.603, P = 0.029)	Decreasing	Exploratory lower Th17-related signal
IL-4	Not significant	Raw-P exploratory decrease (FC = 0.715, P = 0.033)	Decreasing	Exploratory lower Th2-related signal
IL-12p70	Not significant	Raw-P exploratory decrease (FC = 0.618, P = 0.011)	Decreasing	Exploratory lower Th1/NK-related signal
IL-6R	Not significant	Raw-P exploratory decrease (FC = 0.684, P = 0.044)	Decreasing	Exploratory lower IL-6R signal

“Significant upregulation” indicates adj. P.Val <0.05. “Exploratory downregulation” indicates raw P <0.05 without significance after multiple-testing correction. “Not significant” indicates no statistically significant difference between groups. IL-16 data in the K3 versus K1 full table were as follows: AveExp K3 = 13.835, AveExp K1 = 15.117, logFC = −1.282, P Value = 0.114, and adj. P.Val = 0.272.

### Functional enrichment

An exploratory enrichment summary based on the seven raw P K3-versus-K1 proteins is provided only as **Supplementary Figure 1**. Because these proteins did not survive Benjamini–Hochberg correction, the enrichment output was not used to support pathway-level or mechanistic conclusions in the main text.

## Discussion

This pilot study compared SF cytokine profiles among a K–L grade 0 non-radiographic comparator group, early OA, and late OA using a high-throughput antibody array. The principal strengths of the study are the use of synovial fluid, a compartment directly related to the intra-articular immune microenvironment, stage-stratified profiling, and explicit multiple-testing correction. The principal observation was that IL-16 and RANTES/CCL5 were elevated in early OA SF and were the only proteins to survive Benjamini–Hochberg correction in the array-based K1 versus K0 comparison. This finding identifies preliminary pilot array-based inflammatory signals for follow-up, but it does not validate IL-16 or CCL5 as clinical biomarkers.

IL-16 as a hypothesis-generating early-stage signal. IL-16 is a pleiotropic cytokine that recruits CD4+ T cells, monocytes, and eosinophils to inflamed tissues via CD4 (16, 24). The 15.79-fold upregulation observed in early OA SF is biologically plausible in light of Luo et al. (25), who reported IL-16 upregulation in OA cartilage tissue by qRT-PCR and immunohistochemistry and identified IL-16 as a downstream target of novel-miR-81 in OA progression. Cho et al. reported that IL-17 can induce IL-16 production in RA synovial cells (17). However, these references provide biological context only; the present study cannot establish an IL-16 mechanism, pathway, or biomarker role without quantitative validation in a larger cohort.

RANTES/CCL5 and chemokine-related synovial inflammation. CCL5 is a key CC chemokine with chemotactic activity for T cells, monocytes, and eosinophils (18). The 11.83-fold elevation in K1 versus K0 is directionally consistent with prior work linking CCL5/CCR5 signaling and chemokine profiles to synovial inflammation (19, 26–29). Nevertheless, these studies provide biological plausibility rather than mechanistic proof for the present dataset. The current data remain array-based and unvalidated, and the lower mean signal in K3 should be interpreted as a cross-sectional observation rather than proof of a longitudinal or temporal change.

### Exploratory late-stage cytokine signals

The raw P downregulation of seven proteins in K3 versus K1 warrants cautious interpretation. Because these proteins did not survive multiple-testing correction, the data do not support firm conclusions regarding immune exhaustion, Th17 attenuation, late-stage remodeling mechanisms, or temporal cytokine changes. At most, the findings suggest exploratory cross-sectional patterns that may be tested in larger longitudinal cohorts.

The exploratory enrichment analysis is therefore reported as supplementary hypothesis generation only, and no pathway-level mechanism is inferred from the seven raw P proteins.

### Translational implications

The present data support prioritizing IL-16 and CCL5 for validation, but they do not support clinical implementation, diagnostic classification, or staging-biomarker claims. No ELISA, multiplex bead assay, qPCR validation, external validation cohort, or ROC analysis was available. Therefore, sensitivity, specificity, and AUC were not estimated, and the term biomarker should be understood only as a hypothesis-generating observation rather than a validated diagnostic or staging marker.

### Limitations

This study has several important limitations. First, the sample size was extremely small (K0, n = 4; K1, n = 6; K3, n = 6), and no formal *a priori* power calculation was performed because this was designed as a pilot discovery study rather than a confirmatory biomarker validation study. With only four K–L grade 0 comparator samples, variance estimation is unstable and confidence intervals around effect sizes are wide; therefore, the results should be viewed as hypothesis-generating. Approximate uncertainty intervals for the two K1 versus K0 signals are provided in **Supplementary Table 2**. Second, the K0 group should be interpreted as a K–L grade 0 non-radiographic comparator group rather than unequivocally healthy synovial fluid. The clinical indication for K0 aspiration, MRI exclusion of synovitis or meniscal pathology, and detailed effusion or injury history were not available. Third, the cross-sectional design precludes causal inference, temporal disease modeling, and within-individual progression analysis. Fourth, antibody arrays provide semi-quantitative measurements with a limited dynamic range; thus, the method is appropriate for hypothesis generation but not sufficient for quantitative biomarker validation. The lack of ELISA or other orthogonal validation is a major limitation, and independent validation is required before any biomarker claim can be made. Fifth, potential confounders, including age, BMI, medication use, NSAID exposure, metabolic syndrome, smoking status, effusion, prior injury or surgery, and comorbidities, could not be fully adjusted in this small cohort because detailed data were unavailable. Finally, all samples were collected at a single center, limiting external validity. Taken together, these limitations mean that the validity of the present findings is restricted to preliminary signal detection and does not extend to diagnostic accuracy, temporal disease modeling, or clinical decision-making.

### Conclusions

In this pilot high-throughput antibody-array study of synovial fluid, IL-16 and RANTES/CCL5 showed early-OA-associated pilot array-based inflammatory signals after Benjamini–Hochberg correction in the K1 versus K–L grade 0 comparator comparison. K3-related cytokine changes and pathway results were exploratory and did not survive multiple-testing correction. These findings justify targeted follow-up but require validation in larger independent cohorts using quantitative assays before any diagnostic, staging, therapeutic-monitoring, or other clinical application can be proposed.

## Data Availability

The raw data supporting the conclusions of this article will be made available by the authors, without undue reservation.

## Supplementary material

Supplementary Figure 1 Exploratory functional enrichment of raw-P proteins. Gene Ontology (GO) and Kyoto Encyclopedia of Genes and Genomes (KEGG) pathway enrichment analyses were performed on the seven raw-P exploratory proteins from K3 versus K1. Because these proteins did not survive Benjamini-Hochberg correction, the enrichment results should be interpreted only as bioinformatic hypothesis generation and not as mechanistic evidence.
