# Supplementary material for: Synovial fluid IL-16 and RANTES/CCL5 signals in early knee osteoarthritis: a pilot antibody-array study
Source: Front Immunol. 2026 Jul 8;17:1889340. doi: 10.3389/fimmu.2026.1889340 (PMC13388094; doi:10.3389/fimmu.2026.1889340)
Supplement: Supplementary Figure 1 — Exploratory functional enrichment of raw-P proteins. Gene Ontology (GO) and Kyoto Encyclopedia of Genes and Genomes (KEGG) pathway enrichment analyses were performed on the seven raw-P exploratory proteins from K3 versus K1. Because these proteins did not survive Benjamini-Hochberg correction, the enrichment results should be interpreted only as bioinformatic hypothesis generation and not as mechanistic evidence. [file SupplementaryFile1.zip › Supplementary_Material.docx]

**Supplementary Material**

Synovial fluid IL-16 and RANTES/CCL5 signals in early knee osteoarthritis: a pilot antibody-array study

**Supplementary Table S1. Full 40-protein panel of the RayBiotech Human Inflammation Antibody Array (QAH-INF-3).**

| **No.** | **Protein target** | **Alternative name / gene symbol** | **No.** | **Protein target** | **Alternative name / gene symbol** |
| --- | --- | --- | --- | --- | --- |
| 1 | BLC | BCA-1 / CXCL13 | 21 | IL-12 p40 | IL12B |
| 2 | Eotaxin | CCL11 | 22 | IL-12 p70 | IL12A |
| 3 | Eotaxin-2 | MPIF-2 / CCL24 | 23 | IL-13 | IL13 |
| 4 | G-CSF | CSF3 | 24 | IL-15 | IL15 |
| 5 | GM-CSF | CSF2 | 25 | IL-16 | IL16 |
| 6 | I-309 | TCA-3 / CCL1 | 26 | IL-17A | IL17A |
| 7 | ICAM-1 | CD54 / ICAM1 | 27 | MCP-1 | CCL2 |
| 8 | IFN-gamma | IFNG | 28 | M-CSF | CSF1 |
| 9 | IL-1 alpha | IL-1F1 / IL1A | 29 | MIG | CXCL9 |
| 10 | IL-1 beta | IL-1F2 / IL1B | 30 | MIP-1 alpha | CCL3 |
| 11 | IL-1Ra | IL-1RN / IL-1F3 / IRAP / IL1RN | 31 | MIP-1 beta | CCL4 |
| 12 | IL-2 | IL2 | 32 | MIP-1 delta | CCL15 |
| 13 | IL-4 | IL4 | 33 | PDGF-BB | PDGFB |
| 14 | IL-5 | IL5 | 34 | RANTES | CCL5 |
| 15 | IL-6 | IL6 | 35 | TIMP-1 | TIMP1 |
| 16 | IL-6R | IL6RA | 36 | TIMP-2 | TIMP2 |
| 17 | IL-7 | IL7 | 37 | TNF alpha | TNFA |
| 18 | IL-8 | IL8 / CXCL8 | 38 | TNF beta | TNFSF1 / LT-alpha / TNFB |
| 19 | IL-10 | IL10 | 39 | TNF R1 | TNFRSF1A |
| 20 | IL-11 | IL11 | 40 | TNF R2 | TNFRSF1B |

The antibody-array panel is listed for transparency. Protein names are shown as reported by the array platform, with commonly used alternative names or gene symbols where applicable.

**Supplementary Table S2. Approximate post hoc uncertainty intervals for the two K1-versus-K0 signals.**

| **Protein** | **AveExpr K1** | **AveExpr K0** | **logFC** | **Approximate interval for logFC** | **Fold change** | **Approximate interval for fold change** | **adj.P.Val** |
| --- | --- | --- | --- | --- | --- | --- | --- |
| IL-16 | 15.12 | 11.14 | 3.98 | 2.21 to 5.75 | 15.79 | 4.64 to 53.74 | 0.027 |
| RANTES/CCL5 | 16.59 | 13.02 | 3.56 | -1.23 to 8.36 | 11.83 | 0.42 to 329.60 | 0.027 |

Intervals are approximate post hoc uncertainty intervals derived for descriptive reporting in this very small pilot cohort. They should be interpreted cautiously and should not be regarded as formal inferential confidence intervals.

**Supplementary Figure S1. Exploratory functional enrichment of raw-P proteins.**


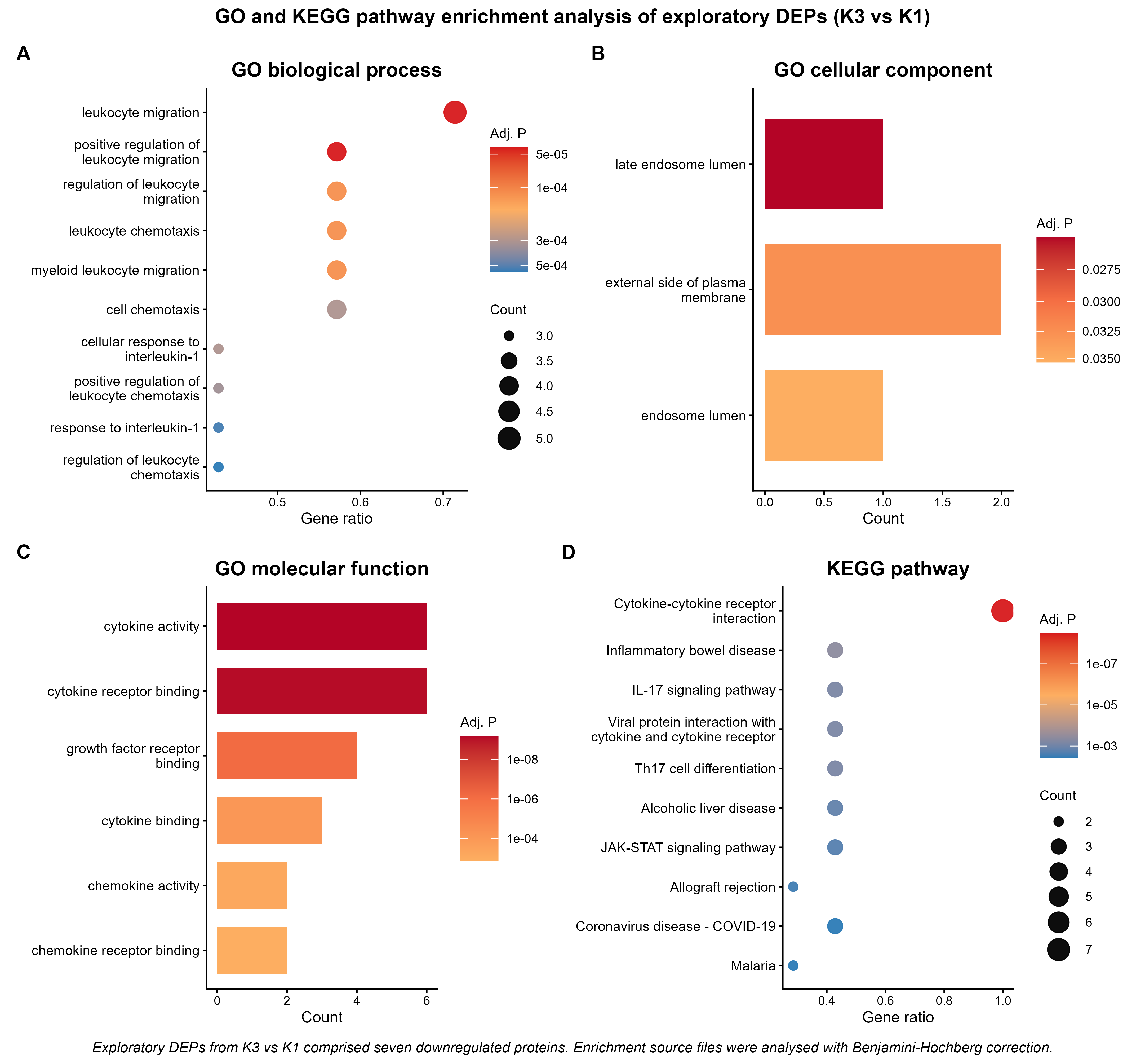


Gene Ontology (GO) and Kyoto Encyclopedia of Genes and Genomes (KEGG) pathway enrichment analyses were performed on the seven raw-P exploratory proteins from K3 versus K1. Because these proteins did not survive Benjamini-Hochberg correction, the enrichment results should be interpreted only as bioinformatic hypothesis generation and not as mechanistic evidence.
