# Supplementary material for: Synovial fluid IL-16 and RANTES/CCL5 signals in early knee osteoarthritis: a pilot antibody-array study
Source: Front Immunol. 2026 Jul 8;17:1889340. doi: 10.3389/fimmu.2026.1889340 (PMC13388094; doi:10.3389/fimmu.2026.1889340)
Supplement: Supplementary Figure 1 — Exploratory functional enrichment of raw-P proteins. Gene Ontology (GO) and Kyoto Encyclopedia of Genes and Genomes (KEGG) pathway enrichment analyses were performed on the seven raw-P exploratory proteins from K3 versus K1. Because these proteins did not survive Benjamini-Hochberg correction, the enrichment results should be interpreted only as bioinformatic hypothesis generation and not as mechanistic evidence. [file SupplementaryFile1.zip › Supplementary_Material.pdf]

Supplementary Material

Synovial fluid IL-16 and RANTES/CCL5 signals in early knee osteoarthritis: a pilot antibody-array study

Supplementary Table S1. Full 40-protein panel of the RayBiotech Human Inflammation Antibody Array (QAH-INF-3).

No.	Protein target	Alternative name / gene symbol	No.	Protein target	Alternative name / gene symbol
1	BLC	BCA-1 / CXCL13	21	IL-12 p40	IL12B
2	Eotaxin	CCL11	22	IL-12 p70	IL12A
3	Eotaxin-2	MPIF-2 / CCL24	23	IL-13	IL13
4	G-CSF	CSF3	24	IL-15	IL15
5	GM-CSF	CSF2	25	IL-16	IL16
6	I-309	TCA-3 / CCL1	26	IL-17A	IL17A
7	ICAM-1	CD54 / ICAM1	27	MCP-1	CCL2
8	IFN-gamma	IFNG	28	M-CSF	CSF1
9	IL-1 alpha	IL-1F1 / IL1A	29	MIG	CXCL9
10	IL-1 beta	IL-1F2 / IL1B	30	MIP-1 alpha	CCL3
11	IL-1Ra	IL-1RN / IL-1F3 / IRAP / IL1RN	31	MIP-1 beta	CCL4
12	IL-2	IL2	32	MIP-1 delta	CCL15
13	IL-4	IL4	33	PDGF-BB	PDGFB
14	IL-5	IL5	34	RANTES	CCL5
15	IL-6	IL6	35	TIMP-1	TIMP1
16	IL-6R	IL6RA	36	TIMP-2	TIMP2
17	IL-7	IL7	37	TNF alpha	TNFA
18	IL-8	IL8 / CXCL8	38	TNF beta	TNFSF1 / LT-alpha / TNFB
19	IL-10	IL10	39	TNF R1	TNFRSF1A
20	IL-11	IL11	40	TNF R2	TNFRSF1B

The antibody-array panel is listed for transparency. Protein names are shown as reported by the array platform, with commonly used alternative names or gene symbols where applicable.

Supplementary Table S2. Approximate post hoc uncertainty intervals for the two K1-versus-K0 signals.

Protein	AveExpr K1	AveExpr K0	logFC	Approximate interval for logFC	Fold change	Approximate interval for fold change	adj.P.Val
IL-16	15.12	11.14	3.98	2.21 to 5.75	15.79	4.64 to 53.74	0.027
RANTES/CCL5	16.59	13.02	3.56	-1.23 to 8.36	11.83	0.42 to 329.60	0.027

Intervals are approximate post hoc uncertainty intervals derived for descriptive reporting in this very small pilot cohort. They should be interpreted cautiously and should not be regarded as formal inferential confidence intervals.

Supplementary Figure S1. Exploratory functional enrichment of raw-P proteins.

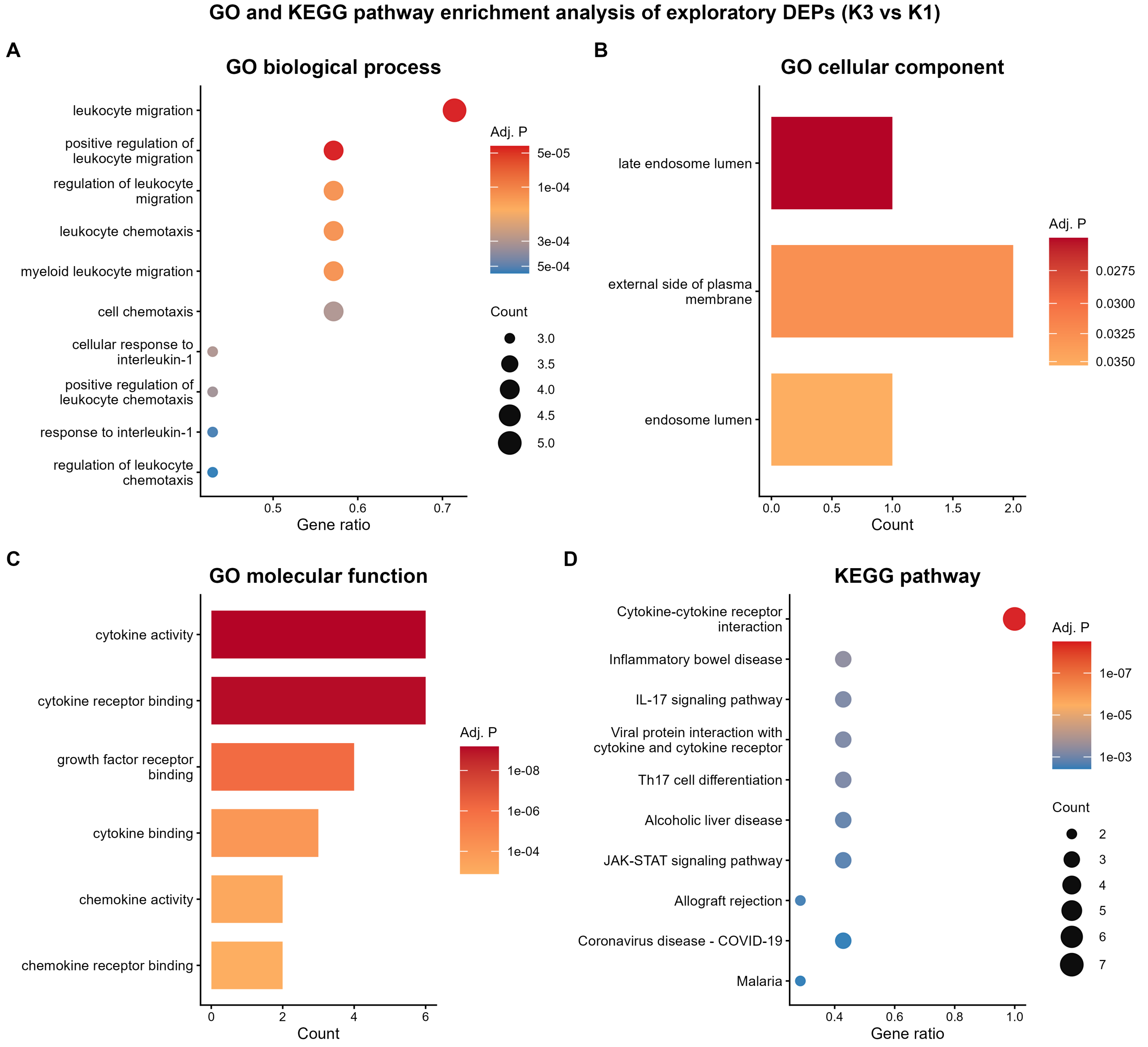

Exploratory DEPs from K3 vs K1 comprised seven downregulated proteins. Enrichment source files were analysed with Benjamini-Hochberg correction.

Gene Ontology (GO) and Kyoto Encyclopedia of Genes and Genomes (KEGG) pathway enrichment analyses were performed on the seven raw-P exploratory proteins from K3 versus K1. Because these proteins did not survive Benjamini-Hochberg correction, the enrichment results should be interpreted only as bioinformatic hypothesis generation and not as mechanistic evidence.
