# Supplementary figures and images for: Synovial fluid IL-16 and RANTES/CCL5 signals in early knee osteoarthritis: a pilot antibody-array study
Source: Front Immunol. 2026 Jul 8;17:1889340. doi: 10.3389/fimmu.2026.1889340 (PMC13388094; doi:10.3389/fimmu.2026.1889340)

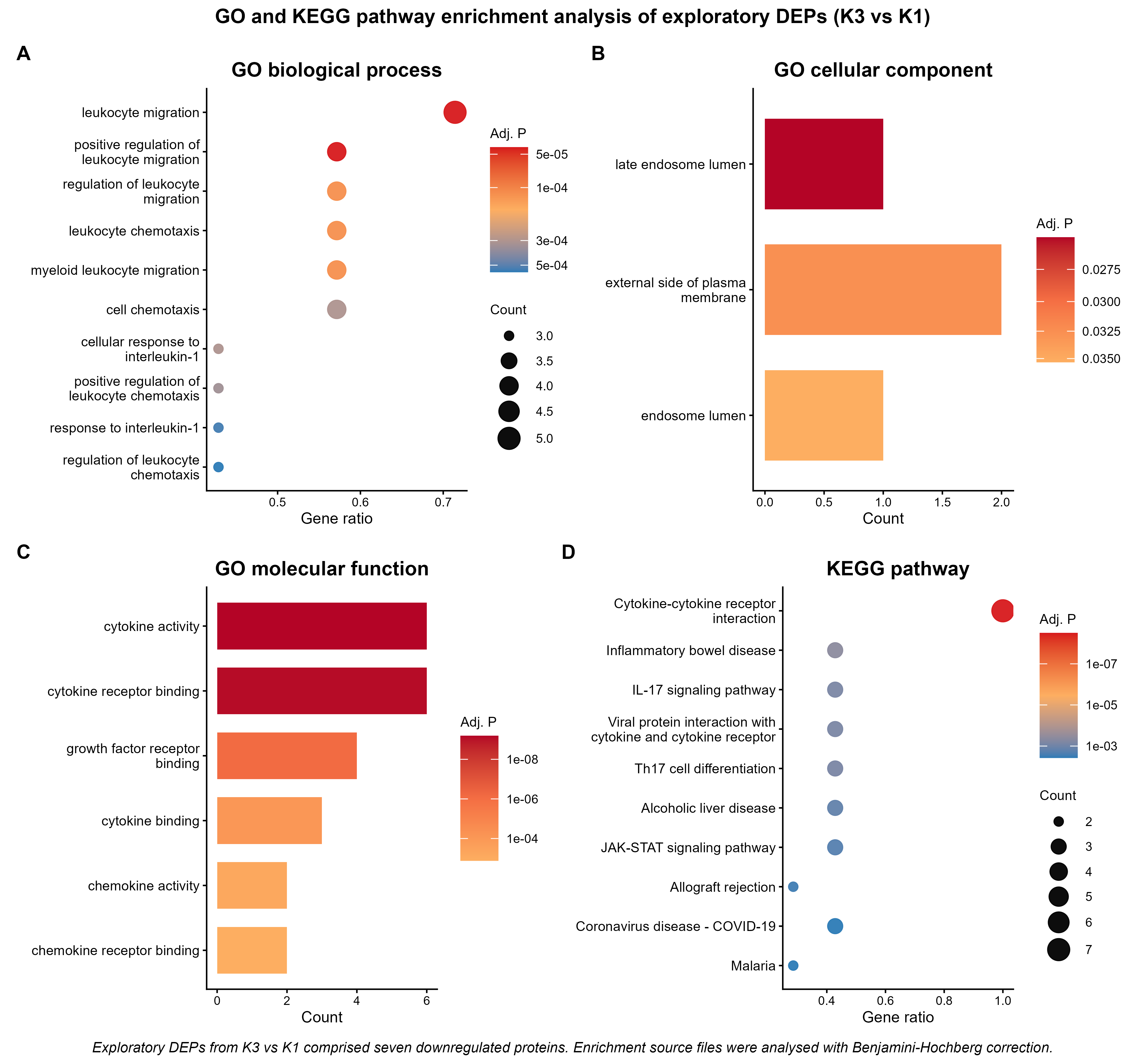

Supplement: Supplementary Figure 1 — Exploratory functional enrichment of raw-P proteins. Gene Ontology (GO) and Kyoto Encyclopedia of Genes and Genomes (KEGG) pathway enrichment analyses were performed on the seven raw-P exploratory proteins from K3 versus K1. Because these proteins did not survive Benjamini-Hochberg correction, the enrichment results should be interpreted only as bioinformatic hypothesis generation and not as mechanistic evidence. [file SupplementaryFile1.zip › Supplementary_Figure_S1.png]
